# Optimization of the composition of temperature-responsive polymers for spin columns

**DOI:** 10.1038/s41598-026-39613-1

**Published:** 2026-02-12

**Authors:** Kenichi Nagase, Matsurika Kokubun, Hideko Kanazawa

**Affiliations:** 1 Graduate School of Biomedical and Health Sciences, Hiroshima University, 1-2-3 Kasumi, Minami-ku, 734-8553 Hiroshima City, Hiroshima Japan; 2 Faculty of Pharmacy, Keio University, 1-5-30 Shibakoen, 105-8512 Minato, Tokyo Japan

**Keywords:** Therapeutic drug monitoring, Temperature-responsive polymer, Temperature-responsive chromatography, Bioseparation, Sample preparation, Chemistry, Drug discovery, Materials science

## Abstract

A straightforward method for deproteinizing serum samples is essential for treatment monitoring. In this study, a temperature-responsive spin column composed of various poly(*N*-isopropylacrylamide) (PNIPAAm) copolymer-modified bead-packed layers was developed. The optimal conditions for separating voriconazole, lamotrigine, and carbamazepine from the serum using the prepared columns were determined. Voriconazole was effectively separated using a bead-packed spin column modified with P(NIPAAm-*co*-*N*,*N*-dimethylacrylamide (DMAPAAm)7.5%-*co*-*n*-butyl methacrylate (BMA)0%) as the upper layer, which was attributed to the desorption of voriconazole at low temperatures. A high elution ratio was achieved for lamotrigine using a bead-packed spin column modified with P(NIPAAm-*co*-DMAPAAm7.5%-*co*-BMA0%). Conversely, a high carbamazepine elution ratio was obtained using P(NIPAAm-*co*-DMAPAAm7.5%–*co*-BMA5%). These findings indicate that the copolymer composition of temperature-responsive spin columns can be tailored for the separation of specific drugs.

**Supplementary Information:**

The online version contains supplementary material available at 10.1038/s41598-026-39613-1.

## Introduction

Therapeutic drug monitoring (TDM) is an effective approach for tailoring drug dosages to individual patients^1,2^. High-performance liquid chromatography (HPLC) is a commonly employed and versatile technique for TDM analysis. However, prior to HPLC analysis, serum proteins must be eliminated from drug samples using methods such as organic solvent extraction, precipitation, or trapping in solid extraction columns or spin columns^3^. Despite this, the use of organic solvents is generally not preferred in hospital environments, highlighting the need for a simple, organic-solvent-free method to remove serum proteins.

Temperature-responsive chromatography using poly(*N*-isopropylacrylamide) (PNIPAAm) has been explored for separation analysis using a completely aqueous mobile phase^4–9^. PNIPAAm undergoes notable changes in hydrophilicity and hydrophobicity owing to hydration and dehydration when exposed to temperature variations^10^. This polymer has been extensively applied in the biomedical field^11–13^, including in systems for temperature-controlled drug and gene delivery^14–19^, biosensors and diagnostic devices^20–26^, materials for cell separation^27–34^, and functional cell culture dishes used in tissue engineering^35–42^. In this type of chromatography, silica beads modified with PNIPAAm serves as the stationary phase, and the hydrophobic interaction between the analyte and PNIPAAm on these beads is adjusted by simply altering the column temperature using an all-aqueous mobile phase. Conversely, traditional reverse-phase chromatography releases analytes from the stationary phase by incorporating organic solvents into the mobile phase.

In temperature-responsive chromatography, the hydrophobic interactions between the stationary phase and the analyte can be intensified by incorporating a hydrophobic monomer, such as *n*-butyl methacrylate (BMA), into PNIPAAm^43–46^. Additionally, by adding an ionic monomer such as *N*,*N*-dimethylaminopropyl acrylamide (DMAPAAm) to PNIPAAm, the electrostatic interaction between the analyte and stationary phase can be modulated by varying the temperature. This allows the PNIPAAm ionic copolymer to either adsorb or release proteins, depending on the temperature^47–49^. If these PNIPAAm copolymers are used in drug sample preparation, serum proteins can be extracted from the sample using an entirely aqueous eluent.

**Fig. 1:**
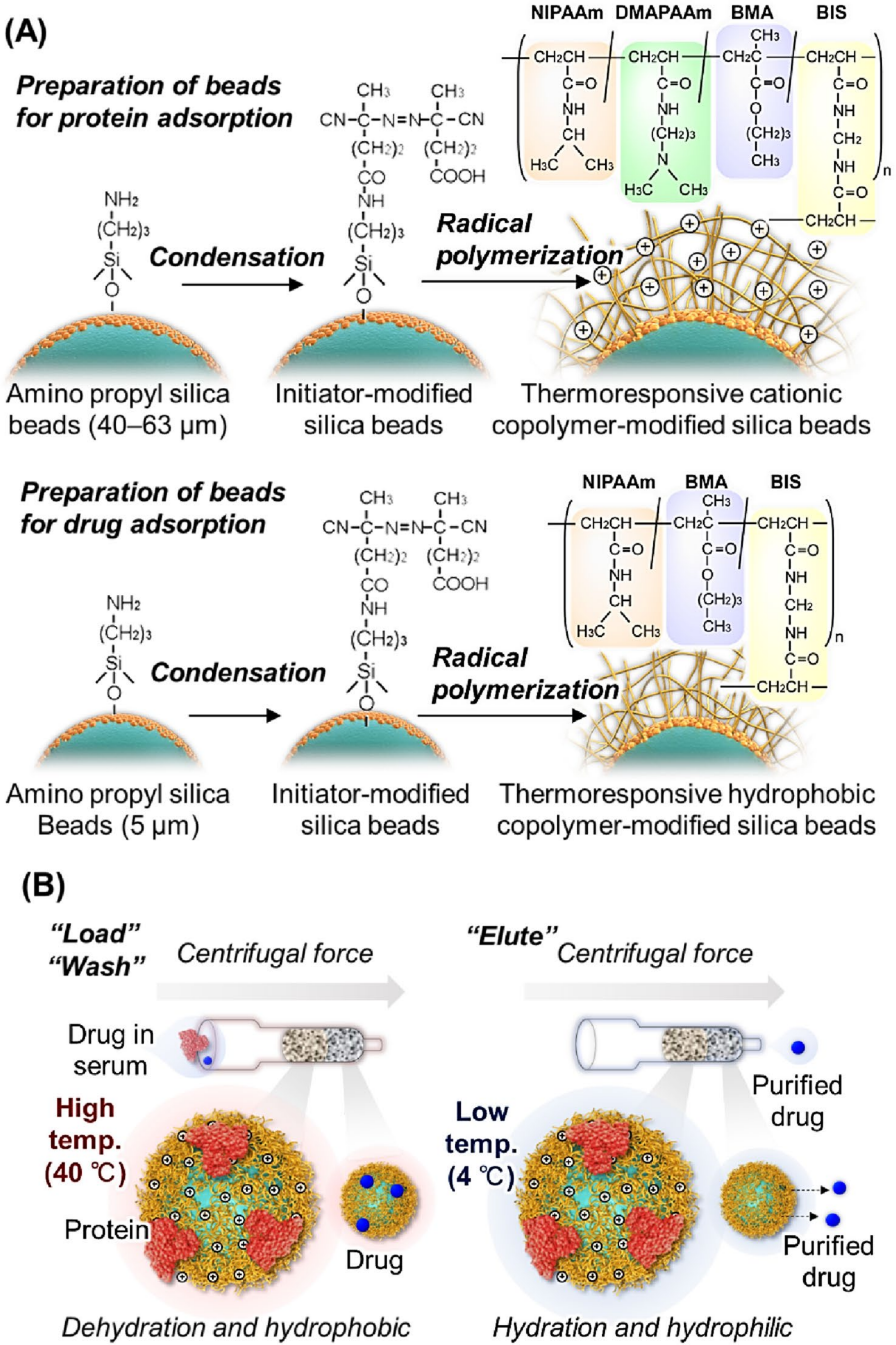
Temperature-responsive spin column for facile sample preparation using an aqueous eluent: (**A**) Preparation scheme of the packing materials in the spin column; (**B**) Schematic of sample preparation in the temperature-responsive spin column.

Therefore, utilizing the properties of this temperature-responsive polymer, we developed a spin column for protein removal from serum and drug samples using aqueous solvents (Fig. 1). At elevated temperatures, serum proteins and drugs adsorb onto the spin columns. Subsequently, lowering the temperature elutes the adsorbed drugs, allowing their recovery from the column. The temperature-responsive spin column consists of two distinct bead layers: the upper layer, packed with larger beads, primarily adsorbs serum proteins, whereas the lower layer, packed with smaller beads, primarily adsorbs drugs. The adsorption and desorption performances of proteins and drugs vary significantly depending on the composition of the temperature-responsive polymer in the spin column. Therefore, determining the optimal polymer composition for each drug is necessary. In this study, we examined polymer compositions suitable for protein removal from serum drug samples, specifically voriconazole, lamotrigine, and carbamazepine.

## Results and discussion

### Characterization of prepared beads

The silica beads were characterized by CHN elemental analysis (Table 1). For both large- and small-particle silica beads, the C and N contents of the V-501–modified silica beads were much higher than those of the aminopropyl silica beads. These results indicated that V-501 was modified via a condensation reaction, with modification amounts of 0.733 and 2.18 µmol/m² for large and small silica beads, respectively. These amounts are equivalent to or exceed those reported in previous studies^50–52^ and indicate that sufficient V-501 was modified on the silica bead surface.

**Table 1:** Characterization of the prepared beads.

Sample		Elemental composition (%)		%C_(calcd)_^b)^	Modiifed initiator (µmol/m^2^)^c)^	Grafted polymer (mg/m^2^)^c)^
		C^a)^	N^a)^			
40–63 μm	Aminopropyl silica beads	1.84 ± 0.15	0.67 ± 0.05			
	V-501–modified beads	5.74 ± 0.12	2.01 ± 0.05	0.514	0.733	
	P(NIPAAm-*co*-DMAPAAm7.5%-*co*-BMA5%)-modified beads	11.1 ± 0.30	2.67 ± 0.07	0.637		0.251
5 μm	Aminopropyl silica beads	2.99 ± 0.01	1.12 ± 0.01			
	V-501–modified beads	11.2 ± 0.02	3.83 ± 0.01	0.514	2.18	
	P(NIPAAm-*co*-BMA7%)-modified beads	15.4 ± 0.09	3.61 ± 0.02	0.637		0.274

a) Determined by CHN elemental analysis; b) calculated as the ratio of the molecular weight of carbon in each monomer to the total molecular weight of each monomer; and c) estimated from the carbon composition.

Furthermore, the C and N contents of the polymer silica beads with large and small particle sizes are higher than those of the corresponding V-501–modified silica beads. The amounts of polymer modification were 0.251 and 0.274 mg/m ² for the large and small silica beads, respectively. These values are comparable to those of temperature-responsive polymers modified by radical polymerization in previous studies^46,53^, indicating that the radical polymerization in this study resulted in the incorporation of an appropriate amount of temperature-responsive polymers.

**Fig. 2:**
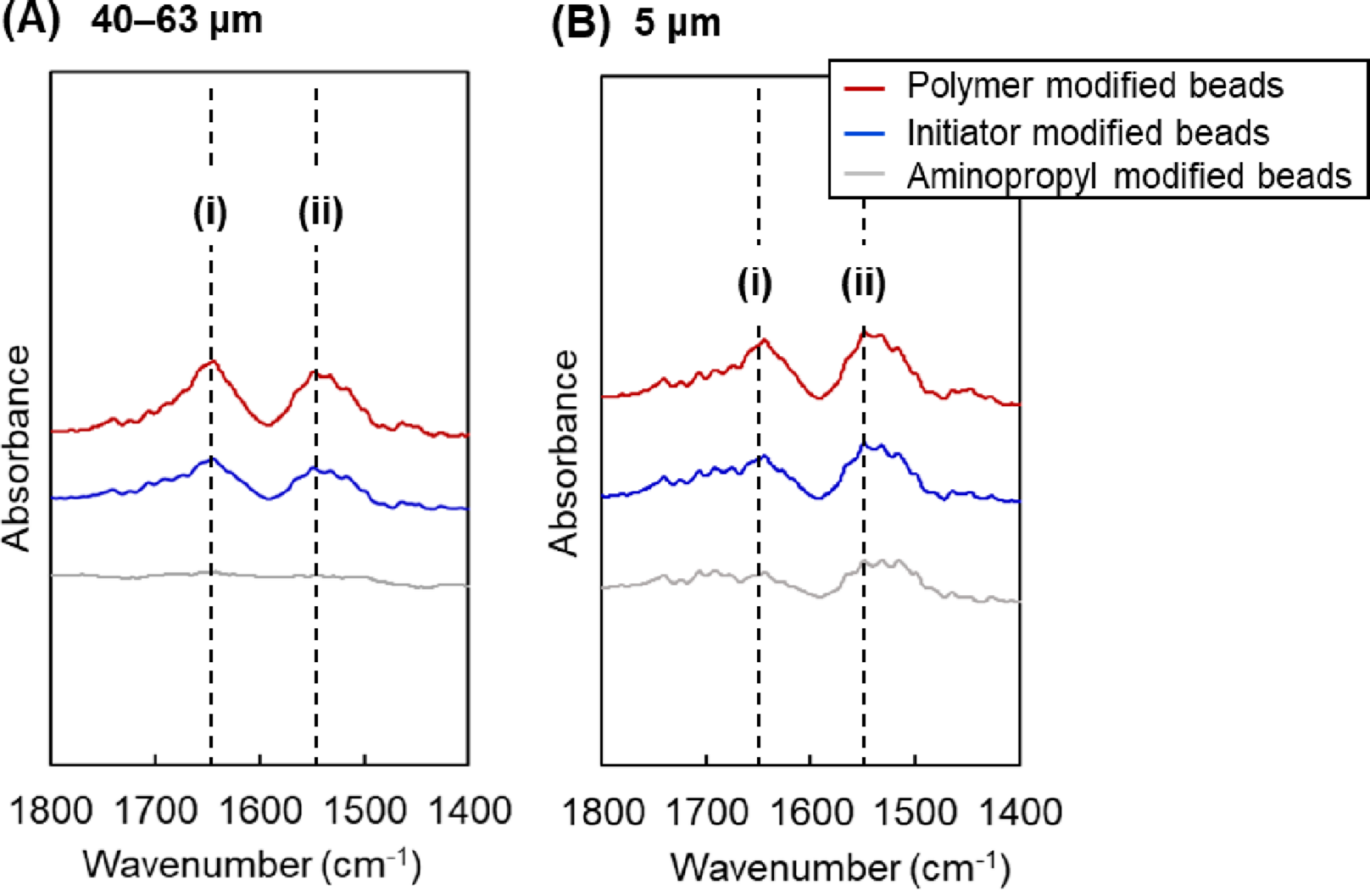
FTIR spectra of the prepared beads. (**A**) Silica beads with diameters of 40–63 μm, (**B**) silica beads with a diameter of 5 μm. The dashed lines (i) and (ii) indicate the peaks attributed to C = O stretching vibrations and N–H bending vibrations, respectively. “Polymer-modified beads” refers to the P(NIPAAm-*co*-DMAPAAm7.5%-*co*-BMA5%)-modified beads for 40–63 μm silica beads, and P(NIPAAm-*co*-BMA7%)-modified beads for 5 μm silica beads.

FTIR spectroscopy was used to analyze the beads at each stage of the reaction. Figure 2 displays the FTIR spectra. In the spectra of beads modified with P(NIPAAm-*co*-DMAPAAm-*co*-BMA) and P(NIPAAm-*co*-BMA), the peaks at 1550 and 1645 cm^− 1^ were assigned to the N–H bending and C=O stretching modes, respectively, corresponding to the N–H and C=O bonds of the modified copolymer on the silica beads. The spectra confirmed that the copolymer was successfully grafted onto the beads through the polymerization reaction.

**Fig. 3:**
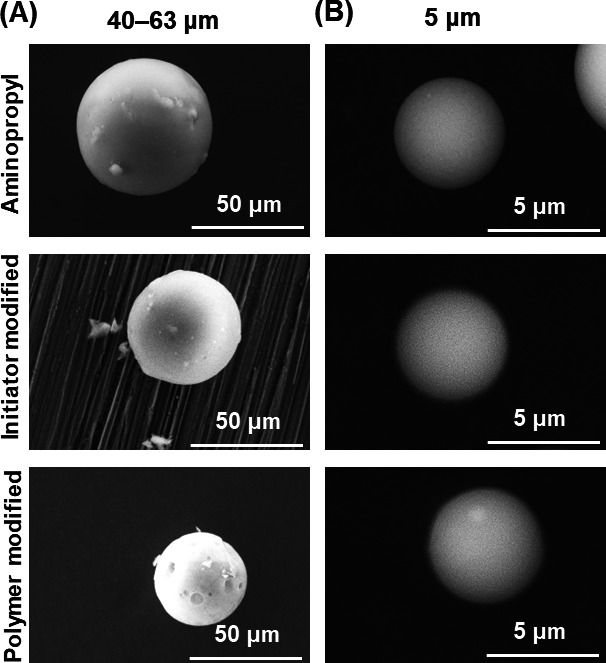
SEM images of the prepared beads. (**A**) Silica beads with a diameter of 40–63 μm and (**B**) silica beads with a diameter of 5 μm. “Polymer modified” refers to the P(NIPAAm-*co*-DMAPAAm7.5%-*co*-BMA5%)-modified beads for 40–63 μm silica beads, and P(NIPAAm-*co*-BMA7%)-modified beads for 5 μm silica beads.

SEM was used to analyze the morphology of the prepared beads (Fig. 3). The beads retained their initial spherical shape following the immobilization of V-501 and polymer modification, indicating that the reaction conditions preserved their integrity. Additionally, the hydrogel-copolymer-modified beads did not aggregate, implying that radical copolymerization of the copolymer on the silica beads was effectively controlled.

**Fig. 4:**
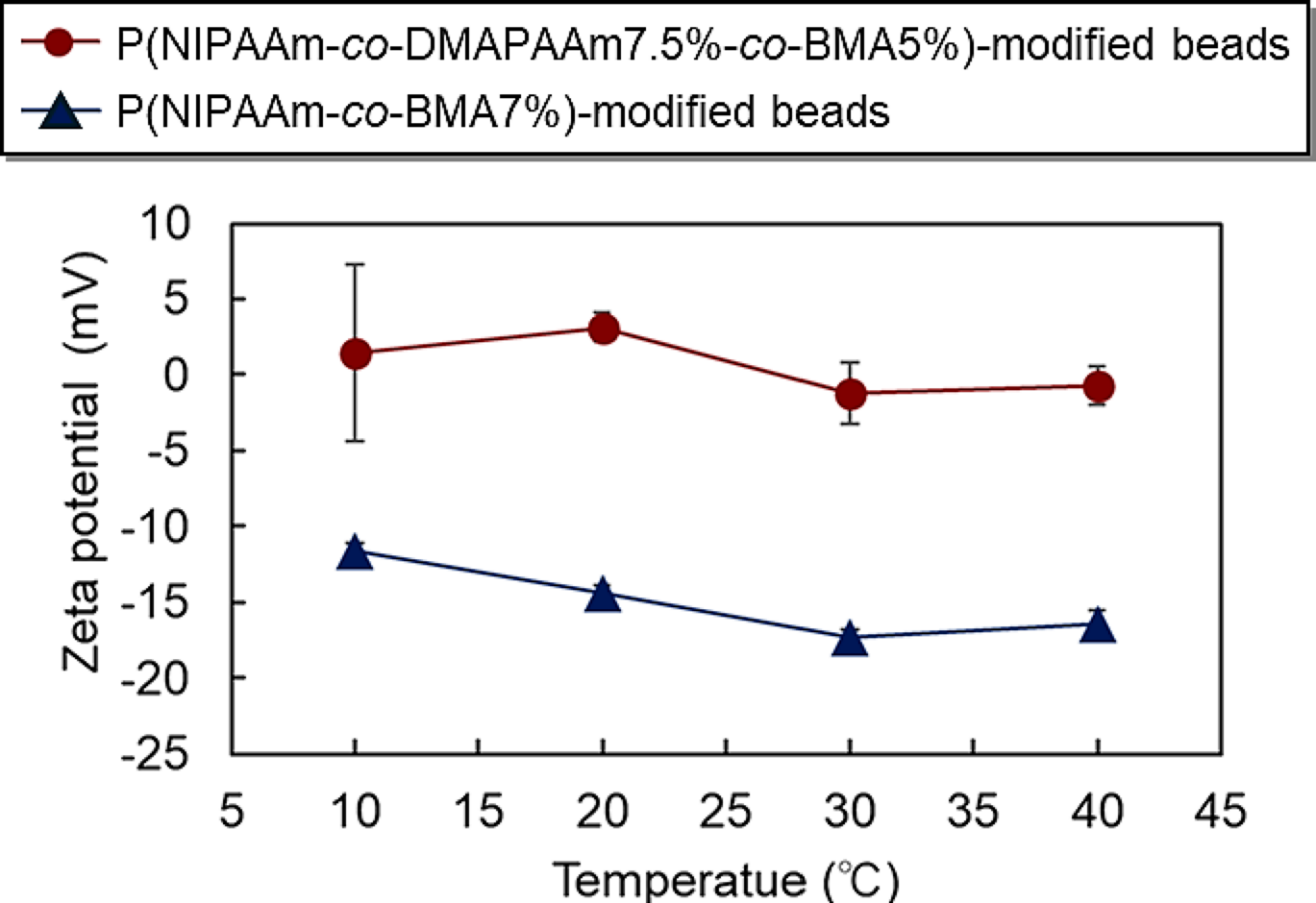
Zeta potential of the prepared copolymer-modified silica beads.

To investigate the surface potentials of the fabricated temperature-responsive polymer-modified beads, their zeta potentials were measured (Fig. 4). The P(NIPAAm-*co*-DMAPAAm7.5%-*co*-BMA5%)-modified silica beads exhibited a higher zeta potential than the P(NIPAAm-*co*-BMA7%)-modified silica beads. This is attributed to the increased zeta potential caused by the cationic DMAPAAm contained within the polymer. By contrast, beads modified with P(NIPAAm-*co*-BMA7%) exhibited a negative zeta potential, despite the polymer being neutral. This is thought to reflect the negative charge inherent in the silanol groups on the silica beads. Both types of polymer-modified silica beads showed a trend of decreasing zeta potential with increasing temperature. This is thought to occur because the polymers contract as the temperature rises, allowing the inherent properties of the silica beads to become more apparent. The lowest measurement temperature for the zeta potential was 10 °C. This is because the minimum temperature setting on the zeta potential measurement instrument was 10 °C.

The lower critical solution temperatures (LCST) of P(NIPAAm-*co*-DMAPAAm7.5%-*co*-BMA5%) and P(NIPAAm-*co*-BMA7%) are important for the temperature modulation of protein and drug adsorption and desorption. In a previous study, the LCST of the copolymers was investigated by transmittance change of the polymer solution with changing temperature^50^. The LCST of the P(NIPAAm-*co*-DMAPAAm7.5%-*co*-BMA5%) and P(NIPAAm-*co*-BMA7%) are 33.0 °C and 7.6 °C, respectively. Thus, these copolymers are suitable for temperature modulation between 40 °C and 4 °C.

### Elution of drug and serum from spin column

The two types of beads were packed in an empty spin column. The P(NIPAAm-*co*-BMA)-modified beads (5 μm) were packed into the column as the bottom packed layer for drug adsorption, and the P(NIPAAm-*co*-DMAPAAm-*co*-BMA)-modified beads (40–63 μm) were used as the upper layer for the adsorption of the serum proteins. The elution of three drugs (Table S2) and serum proteins from the prepared spin column was investigated, with centrifugation at 40 °C (Load and Wash fractions) and 4 °C (Elute fraction).

**Fig. 5:**
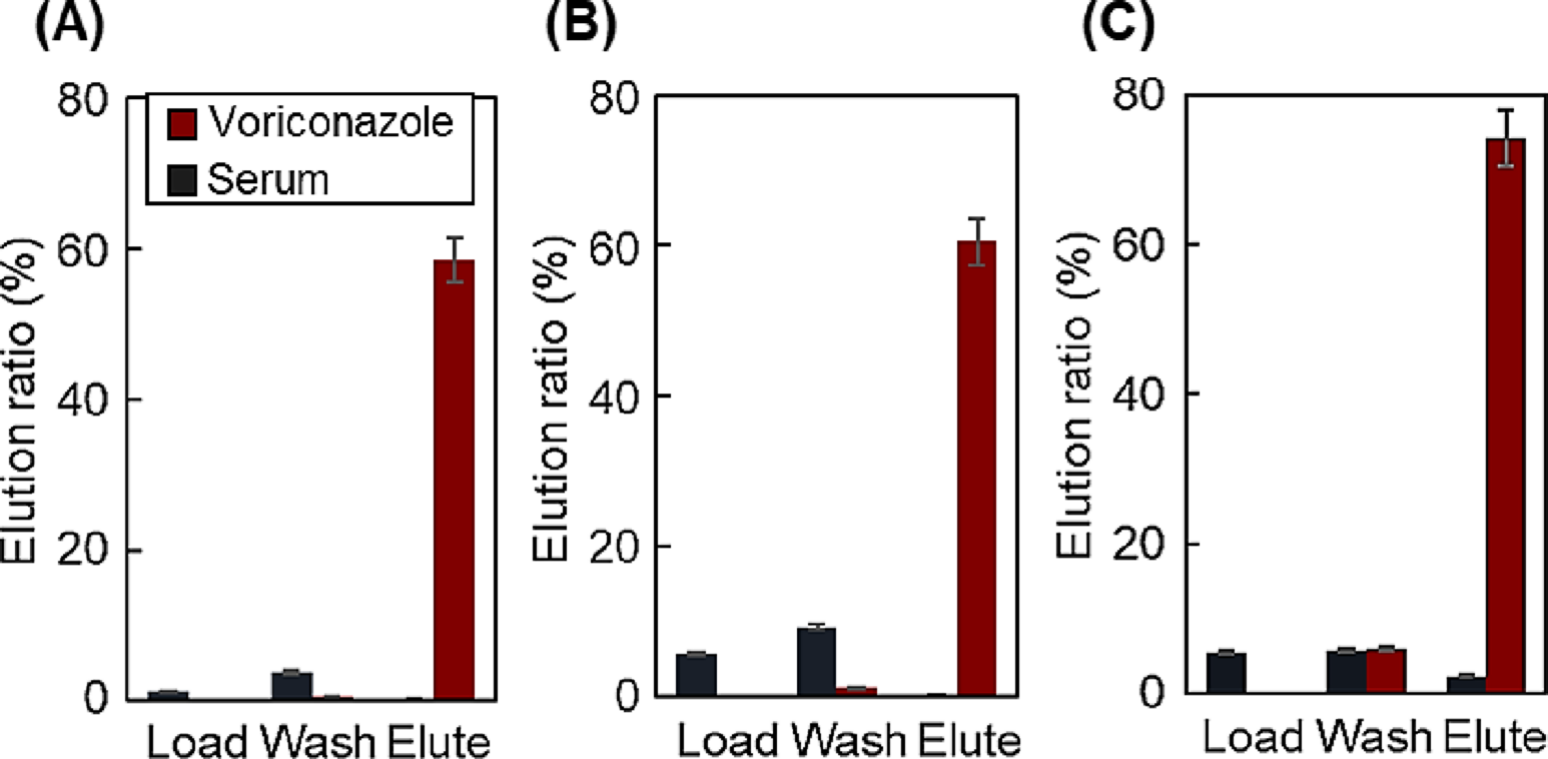
Elution ratios of voriconazole and serum proteins. (**A**) P(NIPAAm-*co*-DMAPAAm7.5%-*co*-BMA5%)-, (**B**) P(NIPAAm-*co*-DMAPAAm10%-*co*-BMA5%)-, and (**C**) P(NIPAAm-*co*-DMAPAAm7.5%-*co*-BMA0%)-modified beads as upper packed layer. The bottom layer of all columns was packed with P(NIPAAm-co-BMA7%)-modified silica beads.

The elution of voriconazole from the spin column was investigated by changing the polymer composition of the upper-packed layer (Fig. 5). The P(NIPAAm-*co*-BMA7%)-modified beads were used as the bottom layers for voriconazole adsorption. Representative chromatograms for obtaining the elution ratio are shown in Supplementary Information Fig. S1. Similar to previous studies, serum samples eluted at short retention times, whereas drugs eluted at relatively long retention times^53,54^. This is because serum proteins do not interact with the P(NIPAAm-*co*-BMA) silica bead-packed column, whereas the drug exhibits strong hydrophobic interactions. The elution ratio was calculated using the peak areas of the serum protein and voriconazole peaks in the obtained chromatogram.

The elution ratio of voriconazole on all columns was low in the Load and Wash fractions at 40 °C because voriconazole was adsorbed onto the copolymer-modified beads through hydrophobic interactions between P(NIPAAm-*co*-BMA7%) and voriconazole. Serum proteins could not be eluted from the column because albumin, the main protein component, was adsorbed onto the P(NIPAAm-*co*-DMAPAAm-*co*-BMA)-modified beads through electrostatic interactions. Adsorbed voriconazole was eluted from the spin column into the Elute fraction at 4 °C because P(NIPAAm-*co*-BMA7%) was hydrated at 4 °C, leading to the adsorption of voriconazole on the copolymer, which was then desorbed and eluted from the column. The elution from the P(NIPAAm-*co*-DMAPAAm7.5%-*co*-BMA0%) column was higher than that from the other columns. The results indicate that the P(NIPAAm-*co*-DMAPAAm-*co*-BMA)-modified beads in the upper layer had a minor influence on the adsorption of voriconazole because the bottom P(NIPAAm-*co*-BMA)-modified bead-packed layer was the same for all columns. P(NIPAAm-*co*-DMAPAAm7.5%-*co*-BMA0%) is less hydrophobic than the other polymers, leading to the elution of the adsorbed voriconazole on P(NIPAAm-*co*-DMAPAAm7.5%-*co*-BMA0%) during the hydration of the copolymer. Thus, voriconazole was effectively purified using the P(NIPAAm-*co*-DMAPAAm7.5%-*co*-BMA0%)-modified beads packed in a spin column as the upper layer.

**Fig. 6:**
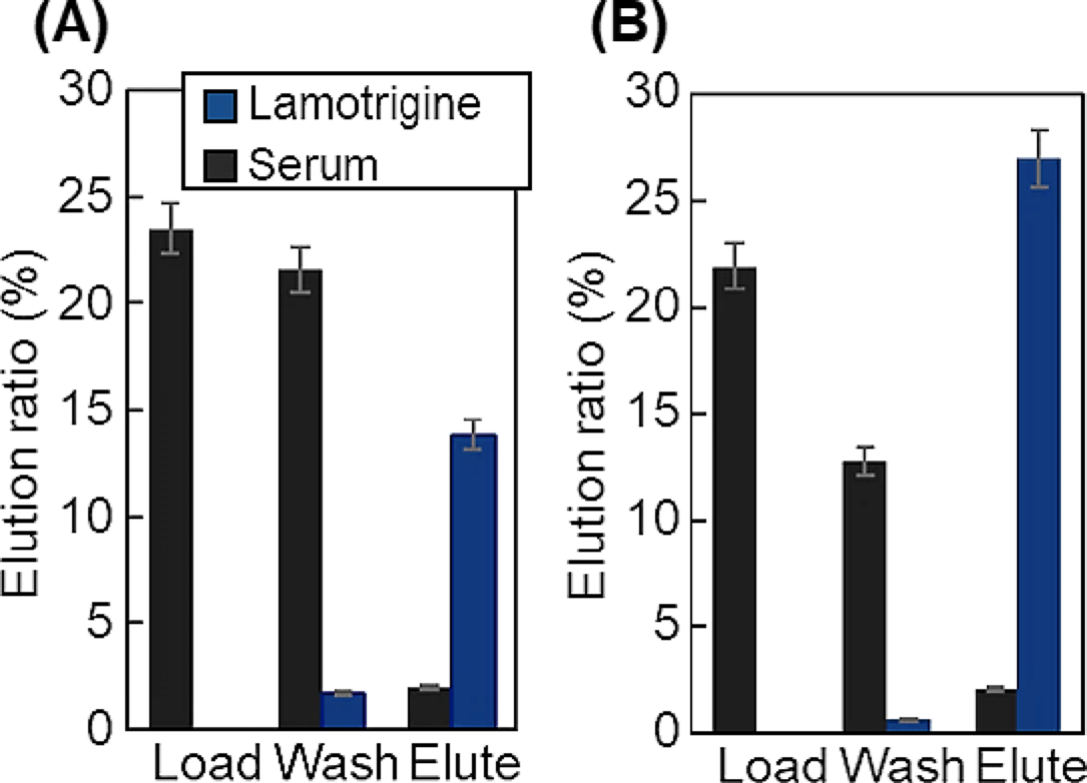
Elution ratios of lamotrigine and serum proteins. (**A**) P(NIPAAm-*co*-DMAPAAm7.5%-*co*-BMA5%)-and (**B**) P(NIPAAm-*co*-DMAPAAm7.5%-*co*-BMA0%)-modified beads were used as the upper packed layer. The bottom layer of all columns was packed with P(NIPAAm-co-BMA7%)-modified silica beads.

The elution of lamotrigine from the prepared spin column was investigated by varying the polymer composition of the upper packed layer (Fig. 6).

The elution ratio of lamotrigine was lower than that of voriconazole owing to the relatively higher hydrophobicity of lamotrigine compared with that of voriconazole. Hydrophobic lamotrigine was adsorbed onto the P(NIPAAm-*co*-BMA7%)-modified silica beads. The spin column composed of P(NIPAAm-*co*-DMAPAAm7.5%-*co*-BMA0%)-modified beads as the upper packed layer provided a relatively higher elution ratio compared with that of the P(NIPAAm-*co*-DMAPAAm7.5%-*co*-BMA5%)-modified bead-packed column. Thus, the P(NIPAAm-*co*-DMAPAAm-*co*-BMA)-modified beads in the upper layer had a minor influence on the adsorption of lamotrigine, as the bottom P(NIPAAm-*co*-BMA)-modified bead-packed layer was identical in all columns. The difference in the elution ratios is attributed to the difference in the hydrophobic properties of the copolymers. The hydrophobicity of P(NIPAAm-*co*-DMAPAAm7.5%-*co*-BMA0%) is lower than that of P(NIPAAm-*co*-DMAPAAm7.5%-*co*-BMA5%). Thus, larger amounts of adsorbed lamotrigine were eluted from the P(NIPAAm-*co*-DMAPAAm7.5%-*co*-BMA0%)-modified beads than from the P(NIPAAm-*co*-DMAPAAm7.5%-*co*-BMA5%)-modified beads. Overall, lamotrigine was effectively purified using P(NIPAAm-*co*-DMAPAAm7.5%-*co*-BMA0%)-modified beads packed in a spin column as the upper layer.

**Fig. 7:**
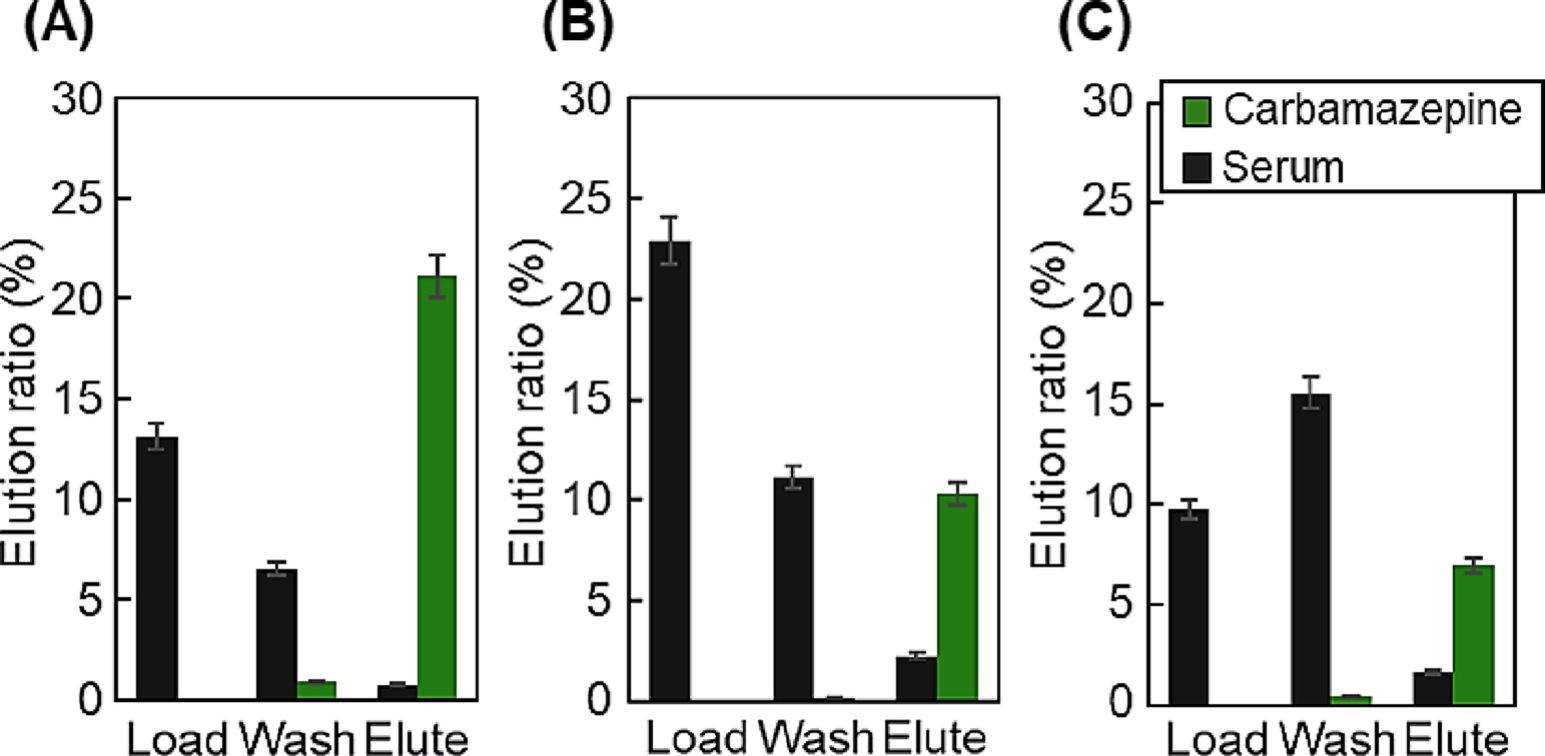
Elution ratios of carbamazepine and serum proteins. (**A**) P(NIPAAm-*co*-DMAPAAm7.5%-*co*-BMA5%)-modified beads as upper layer and P(NIPAAm-*co*-BMA7%)-modified beads as bottom layer, (**B**) P(NIPAAm-*co*-DMAPAAm7.5%-*co*-BMA0%)-modified beads as upper layer and P(NIPAAm-*co*-BMA7%)-modified beads as bottom layer, (**C**) P(NIPAAm-*co*-DMAPAAm7.5%-*co*-BMA5%)-modified beads as upper layer and P(NIPAAm-*co*-BMA3%)-modified beads as bottom layer.

The elution of carbamazepine from the prepared spin column was investigated by varying the polymer compositions of the upper and lower layers (Fig. 7).

Carbamazepine is more hydrophobic than lamotrigine and voriconazole. Thus, the elution ratio of carbamazepine was relatively lower than those of the other drugs because the former strongly adsorbed. Among the three columns, the spin column composed of P(NIPAAm-*co*-DMAPAAm7.5%-*co*-BMA5%)- and P(NIPAAm-*co*-BMA7%)-modified beads as the upper and lower layers, respectively, exhibited the highest elution ratios. The copolymers in the column had a relatively high hydrophobic BMA monomer content. In a previous study, the temperature responsiveness of PNIPAAm copolymers containing ionic monomers was maintained upon the introduction of BMA monomers^55^. Thus, the incorporation of the BMA monomer increased the temperature responsiveness of the copolymer, leading to the effective adsorption and desorption of carbamazepine.

In this study, temperature-responsive polymer-modified beads were synthesized, and spin columns utilizing these beads were fabricated. The elution behaviors of serum proteins and drugs were analyzed using beads with various polymer compositions. The results showed higher elution rates of voriconazole and lamotrigine when the hydrophobic monomer BMA was not incorporated into the modified polymer of the upper-layer beads. In contrast, the use of beads modified with a polymer containing 5% BMA resulted in a higher dissolution rate of carbamazepine. These results indicated that the optimal polymer composition depends on the target drug. Future studies should determine the optimal polymer compositions for various TDM drugs. In addition, this study tested only 40 °C and 4 °C temperatures, but each drug may have optimal adsorption and desorption temperatures. Identifying these temperatures would enable more efficient pretreatment of serum-drug samples.

## Conclusions

A temperature-responsive spin column comprising two types of copolymer-modified beads was developed for effective sample preparation. The optimal conditions for separating voriconazole, lamotrigine, and carbamazepine from the serum using these columns were determined. Voriconazole was effectively separated using P(NIPAAm-*co*-DMAPAAm7.5%-*co*-BMA0%)-modified beads packed in a spin column as the upper layer, which was attributed to the desorption of voriconazole at low temperatures. A high lamotrigine elution ratio was achieved using a P(NIPAAm-*co*-DMAPAAm7.5%-*co*-BMA0%)-modified bead-packed spin column. In contrast, a high carbamazepine elution ratio was achieved using P(NIPAAm-*co*-DMAPAAm7.5%-*co*-BMA5%). Overall, the copolymer compositions of the temperature-responsive spin columns can be tailored for specific drugs.

## Experimental

### Preparation of thermoresponsive packing materials

The specific reagents used in this study are described in the Supplementary Materials. We used two types of silica beads with diameters of 40–63 μm and 5 μm. The thermoresponsive copolymer was modified on silica beads via radical polymerization as follows:

Large-particle silica beads were synthesized by modifying thermoresponsive polymers with cationic functional groups primarily for the adsorption of serum proteins (Fig. 1A). A solution of 4,4’-azobis(4-cyanovaleric acid) (V-501) (3.5 g, 12.5 mmol) and 2-ethoxy-1-ethoxycarbonyl-1,2-dihydroquinoline (6.18 g, 25.0 mmol) was prepared in *N*,*N*-dimethylformamide (50 mL) in a flask. Aminopropyl silica beads (5.0 g) were then suspended in this solution. The suspension was deoxygenated by bubbling with nitrogen gas for 30 min. Subsequently, the flask was sealed, and the condensation reaction was allowed to proceed for 6 h at 25 °C. Upon completion, the beads were filtered, washed with ethanol, and dried at 25 °C under high-vacuum conditions.

NIPAAm, DMAPAAm, and BMA were dissolved in ethanol (20 mL) at varying molar ratios (Table S1). For example, silica beads modified with P(NIPAAm-*co*-DMAPAAm7.5%-*co*-BMA5%), a copolymer consisting of 87.5 mol% NIPAAm, 7.5 mol% DMAPAAm, and 5 mol% BMA; NIPAAm (4.20 g, 37.1 mmol); DMAPAAm (0.497 g, 3.18 mmol); and BMA (0.302 g, 2.12 mmol) were dissolved in 20 mL of ethanol. *N*,* N’*-methylenebis(acrylamide) (BIS; 0.135 g, 0.876 mmol) was added to the solution as a crosslinker. V-501–modified beads (2.0 g) were suspended in the solution, and the bead suspension was deoxygenated by bubbling nitrogen gas for 30 min. The polymerization reaction was conducted at 70 °C for 5 h. After the reaction, the beads were rinsed with ethanol and dried under vacuum. The modified copolymers on the silica beads are denoted P(NIPAAm-*co*-DMAPAAm X%-*co*-BMA Y%), where X and Y are the molar ratios of the monomers in the feed.

Silica beads with small particle sizes were prepared by modifying the beads with strongly hydrophobic thermoresponsive polymers for drug adsorption. NIPAAm and BMA were dissolved in ethanol (20 mL) in varying molar ratios. The amounts of dissolved monomers are listed in Table S1. For example, silica beads modified with P(NIPAAm-*co*-BMA7%), a copolymer comprising 93 mol% NIPAAm and 7 mol% BMA; NIPAAm (4.57 g, 40.4 mmol); and BMA (0.432 g, 3.04 mmol) were dissolved in ethanol (20 mL). BIS (0.135 g, 0.876 mmol) was then added to the solution as a crosslinker. Polymerization and washing of the beads were conducted using the same procedures as for the large-diameter V-501–modified silica beads (2.0 g).

### Characterization of thermoresponsive packing materials

The synthesized beads were characterized by CHN elemental analysis, attenuated total reflection Fourier-transform infrared (ATR/FT-IR) spectroscopy, zeta potential measurements, and scanning electron microscopy. The quantities of modified initiator and polymer on the silica beads were determined by analyzing their carbon contents using CHN elemental analysis (PE-2400, PerkinElmer, Waltham, MA, USA). The amount of initiator (V-501) on the silica beads was calculated as follows:

1 $${\text{Amount of initiator}}=\frac{{\% {C_I}}}{{\% {C_I}(calcd) \times \left( {1 - \% {C_I}/\% {C_I}(calcd)} \right) \times S}}$$

The variable *%C*_*I*_ represents an increase in the carbon composition of V-501–modified silica beads compared with that of the aminopropyl silica beads. Here, *%C*_*I*_ (calcd) denotes the carbon percentage in the V-501 molecules, and *S* refers to the surface area of the beads. The quantity of polymer on the silica beads was determined using the following equation:

2 $${\text{Amount of polymer}}=\frac{{\% {C_P}}}{{\% {C_P}(calcd) \times \left( {1 - \% {C_P}/\% {C_P}(calcd) - \% {C_I}/\% {C_I}(calcd)} \right) \times S}}$$

The percentage increase in the carbon composition (*%C*_*P*_) of the polymer-modified beads was calculated relative to that of V-501–modified silica beads.

Polymer modification on the silica beads was also confirmed using ATR FT-IR spectroscopy (FTIR-4700; JASCO, Tokyo, Japan). Peaks attributed to the C = O stretching and N–H bending vibrations of the polymer were observed in the FT-IR spectrum at approximately 1650 and 1550 cm^-1^, respectively.

The silica bead morphology was observed using scanning electron microscopy (SEM; TM3030Plus, Hitachi High-tech, Tokyo, Japan) after each reaction step. Approximately 1 mg of silica beads was fixed to the sample stage using double-sided tape. SEM images of the silica beads were obtained at an accelerating voltage of 15 kV and magnification of 10,000.

The ionic characteristics of the beads were assessed by measuring their zeta potentials using a zeta potential analyzer (Malvern Instruments, Malvern, UK). The beads were immersed in a 50 mM potassium chloride solution with a concentration of 0.5 mg/mL. The zeta potential of the beads was recorded over a temperature range of 10–40 °C.

### Elution profiles of serum-drug samples from the prepared spin column

Both bead types were packed into an empty spin column to prepare temperature-responsive spin columns. A membrane filter with a pore diameter of 2 μm was placed inside the outlet of the column, which was connected to a column packer that formed the solid-phase extraction column. P(NIPAAm-*co*-BMA)-modified silica beads (5 μm, 20 mg) were suspended in methanol/water (1:1). The bead suspension was poured into a packed column, and the solvent was vacuumed. P(NIPAAm-*co*-DMAPAAm-*co*-BMA)-modified silica beads (40–63 μm, 100 mg) were suspended in 100 mM ammonium acetate solution. The bead suspension was poured into a column packed with P(NIPAAm-*co*-BMA)-modified silica beads, which were then packed onto the P(NIPAAm-*co*-BMA)-modified silica bead layer while vacuuming the solvent. Both packed bead layers were rinsed with 50 mL of pure water. Finally, the beads were vacuum dried.

Serum samples were prepared using a commercially available pooled human serum quality control material (L-Consera^®^ D). The drug was dissolved in tetrahydrofuran (THF) at a concentration of 1.0 mg/mL. Next, 0.15 mL of the drug solution was added to a glass vessel, and THF was evaporated using flowing nitrogen. Then, 1 mL of the serum sample, prepared by dissolving the freeze-dried serum in 3 mL of pure water, was added to voriconazole in a glass vessel. The prepared solution was diluted three times to obtain the final sampling solution.

This study did not involve the collection of new human samples or any interaction with human subjects, and no identifiable personal data were used. Therefore, ethical approval and informed consent were not required.

To load the samples, the prepared drug serum sample was added to the spin column at 40 °C. The spin column was centrifuged at 100 g for 1 min, followed by centrifugation at 1000 g for 1 min. The fraction eluted from the spin column was designated as the “Load.”

Next, pure water (150 µL) was added to the spin column and incubated at 40 °C. The spin column was centrifuged at 100 g 1 min, followed by 1000 g for 1 min. The fraction eluted from the spin column was designated as the “Wash.”

To elute the drug from the spin column, pure water (100 µL) was added to the spin column at 4 °C, and the spin column was centrifuged at 100 g for 1 min, then 2000 × g for 1 min. Pure water was added, and the mixture was centrifuged three times. The fractions eluted from the spin column were designated “Elute” fractions.

The protein and drug concentrations in each fraction were determined using HPLC. The temperature-responsive column packed with P(NIPAAm-*co*-BMA) hydrogel-modified beads was prepared according to a previously published procedure^50,56^. The PNIPAAm copolymer–modified bead-packed column can separate serum and drugs and determine the drug concentration^53^. The elution ratio of proteins was determined as follows: A sample containing only the same amount of serum as that in the serum-drug sample was measured by HPLC, and the obtained peak area in the chromatogram was set as 100% serum elution ratio. Next, each fraction eluted from the spin column was analyzed by HPLC. The elution ratio of serum protein in each fraction was calculated as the ratio of the peak area of serum in the chromatogram of the fraction to that of the 100% serum elution. Serum proteins measured by chromatograms are considered total serum proteins, not just albumin. This is because previous studies have shown that proteins exhibit minimal adsorption to the neutral PNIPAAm copolymer without ionic functional groups and elute with short retention times from the PNIPAAm copolymer column^57–60^. Therefore, it can be assumed that the components of the peaks measured by HPLC represent the total serum proteins. The drug elution ratio was measured in a similar manner. A sample containing only the same amount of drug as that in the serum drug sample was measured using HPLC, and the peak area obtained was set as the 100% elution ratio of the drug. The drug elution ratio in each fraction was calculated as the ratio of the peak area of the drug in the chromatogram of the fraction to that of the 100% drug elution ratio.

## Supplementary Information

Below is the link to the electronic supplementary material.

Supplementary Material 1

## Data Availability

The datasets used and analyzed during the current study available from the corresponding author on reasonable request.
