# Supplementary material for: Optimization of the composition of temperature-responsive polymers for spin columns
Source: Sci Rep. 2026 Feb 12;16:8550. doi: 10.1038/s41598-026-39613-1 (PMC12976355; doi:10.1038/s41598-026-39613-1)
Supplement: Supplementary file 1 — Supplementary Material 1 [file 41598_2026_39613_MOESM1_ESM.docx]

Supplementary Information

**Optimization of the Composition of Temperature-Responsive Polymers for Spin Columns**

Kenichi Nagase*^a,b^, Matsurika Kokubun^b^, Hideko Kanazawa^b^

1. Graduate School of Biomedical and Health Sciences, Hiroshima University, 1-2-3 Kasumi, Minami-ku, Hiroshima City, Hiroshima, 734-8553, Japan
2. Faculty of Pharmacy, Keio University, 1-5-30 Shibakoen, Minato, Tokyo, 105-8512, Japan.

*Corresponding author: (Phone) +81-82-257-5323

(E-mail) nagase@hiroshima-u.ac.jp

**Materials**

*N*-Isopropylacrylamide (NIPAAm) and *N,N*-dimethylaminopropyl acrylamide (DMAPAAm) were provided by KJ Chemicals (Tokyo, Japan). NIPAAm and DMAPAAm were purified by recrystallization from *n*-hexane and distillation, respectively. BMA, *N,N*-methylenebis acrylamide (BIS), 4,4’-azobis(4-cyanovaleric acid)(V-501), n-hexane, ethanol, *N,N*-dimethylformamide (DMF), and ammonium acetate were purchased from Fujifilm Wako Pure Chemical (Osaka, Japan). BMA was purified by distillation. Tetrahydrofuran (THF) was obtained from Kanto Chemical (Tokyo, Japan). 2-Ethoxy-1-ethoxycarbonyl-1,2-dihydroquinoline (EEDQ) was obtained from Sigma-Aldrich (St Louis, MO, USA). Large-diameter silica beads (LC SORB SPW-B-NH2; diameter, 40–63 μm; pore size, 300 Å; specific surface area, 400 m^2^/g) were purchased from Chemco Scientific (Osaka, Japan). Small-diameter silica beads (YMC GEL NH2; diameter, 5 μm; pore size, 120 Å; specific surface area, 310 m^2^/g) were obtained from YMC (Kyoto, Japan). An empty spin column (Micro Bio-Spin Columns; length, 30 mm; bead volume, 0.8 mL) was purchased from Bio-Rad (Hercules, CA, USA). Voriconazole and lamotrigine were obtained from Tokyo Chemical Industry (Tokyo, Japan). Carbamazepine was obtained from Fujifilm Wako Pure Chemical Corporation (Osaka, Japan). Freeze–dried serum was obtained from Nissui Pharmaceutical Co. (Tokyo, Japan).

**Supplementary Table S1.** Amounts of monomers in polymerization

| Polymer | NIPAAm | | DMAPAAm | | BMA | |
| --- | --- | --- | --- | --- | --- | --- |
|  | (g) | (mmol) | (g) | (mmol) | (g) | (mmol) |
| P(NIPAAm-*co*-DMAPAAm7.5%-*co*-BMA5%) | 4.20 | 37.1 | 0.497 | 3.18 | 0.302 | 2.12 |
| P(NIPAAm-*co*-DMAPAAm7.5%-*co*-BMA5%) | 4.44 | 39.2 | 0.497 | 3.18 | 0 | 0 |
| P(NIPAAm-*co*-DMAPAAm10%-*co*-BMA0%) | 4.04 | 35.7 | 0.657 | 4.20 | 0.299 | 2.10 |
| P(NIPAAm-*co*-BMA7%) | 4.57 | 40.4 | 0 | 0 | 0.432 | 3.04 |
| P(NIPAAm-*co*-BMA3%) | 4.77 | 42.1 | 0 | 0 | 0.185 | 1.30 |

| Drug | Structure | Molecular weight | Log*P* | p*K*_a_ |
| --- | --- | --- | --- | --- |
| Voriconazole |  | 349.31 | 1.0 | 1.63 |
| Lamotrigine | 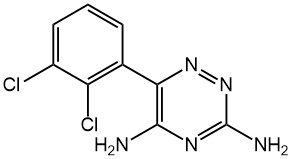 | 256.09 | 2.57 | 5.70 |
| Carbamazepine | 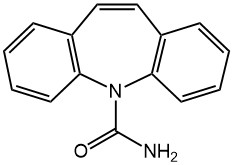 | 236.27 | 2.45 | 13.9 |

**Supplementary Table S2** Properties of drugs in therapeutic drug monitoring


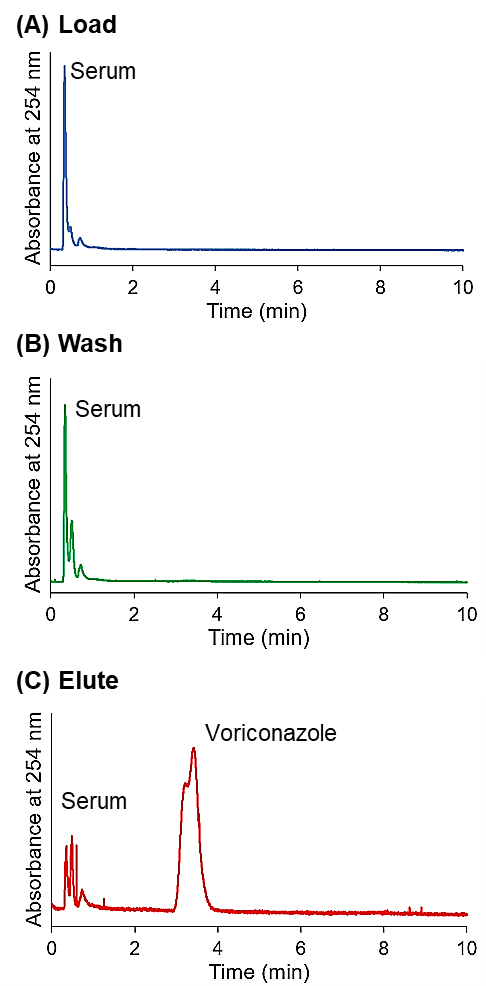


**Supplementary Figure S1** Representative chromatograms for obtaining the elution ratio. Chromatograms were obtained using a P(NIPAAm-*co*-BMA) hydrogel-modified bead-packed column. The mobile phase was 10 mM ammonium acetate with a flow rate of 1.0 mL/min.
